# CTX-M-Producing Bacteria Isolated from a Highly Polluted River System in Portugal

**DOI:** 10.3390/ijerph191911858

**Published:** 2022-09-20

**Authors:** Marta Tacão, José Laço, Pedro Teixeira, Isabel Henriques

**Affiliations:** 1 CESAM and Biology Department, University of Aveiro, 3810-193 Aveiro, Portugal; 2 Department of Life Sciences, University of Coimbra, 3000-456 Coimbra, Portugal; 3 Department of Life Sciences and CFE, University of Coimbra, 3000-456 Coimbra, Portugal

**Keywords:** *bla*_CTX-M_, river pollution, antibiotic resistance, β-lactamases, *Enterobacteriaceae*, pig farms

## Abstract

*Enterobacteriaceae* resistant to third-generation cephalosporins are a great concern for public health, as these are first-line drugs to treat infections. The production of carbapenemases and extended spectrum beta-lactamases (ESBLs) and/or the overexpression of AmpC β-lactamases are the main mechanisms of resistance to these antibiotics. Among the ESBLs, CTX-M β-lactamases are the most prevalent worldwide. Our aims were to determine the prevalence of cefotaxime-resistant *Enterobacteriaceae* along a heavily polluted river and characterize *bla*_CTX-M_ carriers. River water was collected in 11 sites along the main course and tributaries, in two sampling moments. Water quality was evaluated and a collection of cefotaxime-resistant isolates was obtained. *bla*_CTX-M_ carriers were characterized regarding phylogenetic affiliation, clonality, antibiotic susceptibility, gene diversity, and context. Water presented very low quality in all sites. From 147 cefotaxime-resistant isolates, 46% carried *bla*_CTX-M_ and were affiliated with *Escherichia*, *Klebsiella*, *Enterobacter*, and *Citrobacter*. Molecular typing revealed clonal isolates in different sites and over the two years, suggesting survival of the strains in the river or continuous pollution inputs from the same sources. Eight variants of *bla*_CTX-M_ were found, with *bla*_CTX-M-15_ being the most prevalent (52.5%). Sites with a lower water quality showed the highest resistance rates and prevalence of *bla*_CTX-M_, suggesting that river water may embody human health risks.

## 1. Introduction

The pollution of aquatic systems is a concerning issue of global proportions and it has been estimated that more than half of the water resources in the world are polluted [1,2]. Natural processes, such as precipitation and erosion, as well as anthropogenic impacts (urban, industrial, and agriculture origins) and intensive exploration of hydric resources, determine the surface water quality of these systems [3,4,5]. Rivers are among the most intensively human influenced ecosystems worldwide [6]. The accumulation of pollutants in water systems can lead to serious contamination problems with long-term effects on aquatic life and human health. Hence, in view of the diverse water usage purposes and management requirements, assessing water quality is crucial. Consequently, many indices and multivariate models have been proposed to measure surface water quality, none of which considers parameters related to antibiotic resistance [7,8,9].

Antibiotic-resistant bacteria (ARB) and antibiotic-resistant genes (ARGs) are a growing concern in surface waters [10,11,12]. Though naturally present in ecosystems, ARB and ARGs can enter aquatic environments through discharges of untreated water from different sources (e.g., agriculture and industry) and from discharges from wastewater treatment systems, which are considered a major source of these contaminants [10,11,12,13,14,15]. Mixtures of different types of pollutants reaching aquatic settings continuously (e.g., antibiotics, disinfectants, and metals) potentiate the transfer of ARGs between natural and incoming bacterial populations, unbalancing the ecosystem and propagating antibiotic resistance [10,11,12,16]. Hence, with the high levels of contaminants that occur in many rivers worldwide, these environmental compartments can act as hotspots for dispersion of ARGs and ARB.

Broad spectrum antibiotics such as third-generation cephalosporins are very relevant therapeutic agents, as they are the first-line drugs to treat infections caused by multi-resistant gram negative strains, including priority pathogens, such as some of those referred to as ESKAPE [17,18,19]. The consumption of these antibiotics in the European Union is preferentially in human medicine, although relevant amounts are used in veterinary or animal production [20]. Resistance to third-generation cephalosporins in gram negative bacteria is mediated mainly by extended spectrum β-lactamases (ESBLs), which confer resistance to all β-lactams, except to carbapenems and cephamycins, and are currently among the most globally dispersed antibiotic resistance mechanisms [17,21,22]. First identified in 1983 [23], these enzymes are now disseminated worldwide, mainly in gram negatives, showing a significant spread in every World Health Organization (WHO) geographic region [17,21,22,24]. CTX-M type β-lactamases are a huge success in terms of antibiotic resistance dispersion, currently being the most prevalent among ESBLs worldwide [25,26,27]. This dispersion is even more evident when considering only developed countries, reflected by the significant upward trend in Europe [25]. The *bla*_CTX-M_ gene has been linked to highly successful plasmids and high-risk clones, and associated with mechanisms of co-resistance [17,26,28,29]. Even though the majority of β-lactamase genes have an unclear origin, the ancestor of the *bla*_CTX-M_ gene is known to be present in the genome of species of the genus *Kluyvera*, belonging to the *Enterobacteriaceae* family [30,31,32,33]. There have been over 200 different variants of CTX-M enzymes detected so far (https://www.ncbi.nlm.nih.gov/pathogens/refgene/#blaCTX-M; last accessed 31 January 2022). These are divided into six groups: CTX-M-1, CTX-M-2, CTX-M-8, CTX-M-9, CTX-M-25, and KLU-like [21,26], where members of the same group have >94% amino acid identity and there is ≤90% identity across different groups [22]. Worldwide, the most common variants are CTX-M-14 and CTX-M-15, followed by CTX-M-1, CTX-M-2, and CTX-M-3 [25]. Though the vast majority of reports of ARB carrying CTX-M encoding genes refer to clinical isolates, CTX-M producers have been identified in all One Health compartments and interfaces [16,26].

Lis River (Central Portugal) is an example of a highly polluted aquatic system subjected to successive discharges of effluents over its course, which result in a decrease in water quality [34]. Three wastewater treatment plants (WWTPs) discharge their final effluents in this watercourse. Additionally, there are around 400,000 pigs in the Leiria region that produce waste equivalent to that of 1.2 million people [34]. Illegal discharges of piggery wastewater were reported to have a great impact on the water quality in the region, with values of fecal coliforms in the river reaching levels lower than the allowable limits for bathing waters [34,35]. Furthermore, urban effluents, untreated sewage, and agriculture also carry to the river degradable organics, nutrients, and pathogenic organisms [34]. Other contaminants from anthropogenic sources, such as metals, mainly Zn and Mn [36], and antibiotics, e.g., sulfamethoxazole, clarithromycin, and azithromycin [37], were also detected in the river. A recent study detected several clinically relevant *Enterobacteriaceae* resistant to carbapenems (last-resort antibiotics) in the Lis River, carrying clinically important ARGs and having been associated with hospital outbreaks worldwide, including Portugal [38,39]. Therefore, as it receives a variety of contaminants from different sources, this hydrographic system has the potential to serve as a reservoir and a reactor for the evolution and dispersion of ARB and ARGs. Thus, studies developed in this system can contribute to better understanding the role of rivers in the dispersion of resistance and, in general terms, the ecology of antibiotic resistance. Though poor water quality has been documented for decades in this hydrographic basin, including reports on serious ecological disasters in some particular sites [34,35], Lis River water usage in this region is quite important. For example, for irrigation, with agriculture as a key economic activity, or for leisure activities, as the river flows to the Atlantic ocean into busy beach areas. As such, this study had the following intentions: (1) to determine the prevalence of cefotaxime-resistant bacteria along the Lis River, in two consecutive years; (2) to determine the phylogenetic affiliation, clonality, and antibiotic susceptibility profiles of *bla*_CTX-M_ carriers isolated from the Lis River; and (3) to characterize the diversity of *bla*_CTX-M_ genes and their genomic context. For this purpose, water was collected along the Lis River from the spring to the mouth, in the river’s main course and tributaries, considering the location of probable contributors to water pollution in this hydrographic basin. Water quality and the prevalence of cefotaxime-resistant *Enterobacteriaceae* were determined. A collection of cefotaxime-resistant isolates was established. The *bla*_CTX-M_ carriers were characterized regarding their phylogenetic affiliation, clonality, antibiotic resistance profiles, gene diversity, and context. 

## 2. Materials and Methods

### 2.1. Study Area

**Figure 1:**
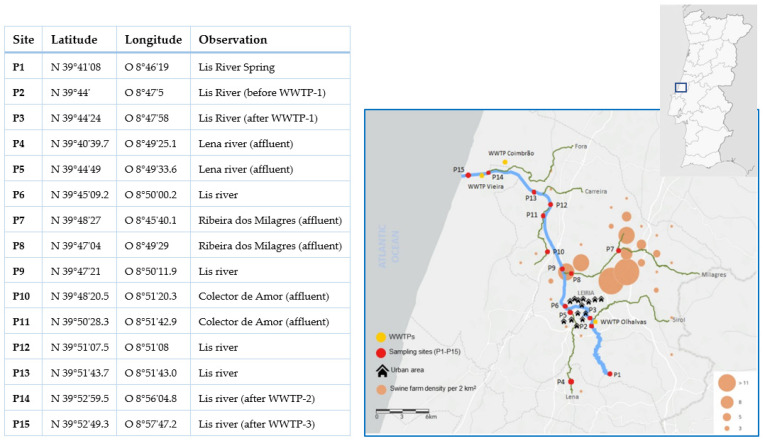
Sampling sites (P1–P15), coordinates, and other information and map showing sites (red dots) located in the Lis River main course (P1–P3, P6, P9, P12–P15) and tributaries (P4, P5, P7, P8, P10). WWTPs are indicated using yellow dots and the density of swine farms is in orange (from Teixeira et al., 2022 [39]).

The hydrographic basin of the Lis River is a coastal basin that comprises an area of approximately 850 km^2^ (Figure 1). The Lis River spring is located at an altitude of 500 m and the river flows into the Atlantic Ocean for a distance of approximately 40 km, with an annual average current of 2.69 m^3^/s. This hydrographic basin is entirely located on soils of the Western Mesocenozoic rim. The annual average temperature and precipitation in this area are 14.8 °C and 855 mm, respectively [34]. The surface water in the main course and its tributaries have a decades-long record of poor water quality owing to the contamination with domestic effluents, agricultural run-offs, and piggery wastewater [34,35,36,37,38,39].

### 2.2. Sampling, Water Quality Evaluation, and Cefotaxime-Resistant Bacteria Isolation

Water samples were collected in September of two consecutive years, 2018 and 2019, from the Lis River (Central Portugal). A total of fifteen sites were analysed, 11 distributed along the main river from the spring to the mouth (P1–P3, P6, P9, P12–P15) and 6 located in three of its affluents (P4, P5, P7, P8, P10, P11). For this selection, we considered urban and rural sites as the location of the wastewater treatment plants (three in the region) and the piggeries facilities, which are considered the main polluters in this hydrographic basin (Figure 1; [39]). Water was collected in sterile bottles and kept on ice for transportation. Physical/chemical parameters were measured three times on site, including pH, temperature, conductivity, and oxygen saturation, using a portable multi-log environmental meter (WTW, Germany). Additional water quality parameters were evaluated in triplicates by collecting 5 L samples, which were sent to a credited laboratory for further analysis, namely of nitrates (ion chromatography), biochemical oxygen demand (gauge), and phosphorus (spectrophotometry). The microbiological parameters evaluated included enterococci, faecal, and total coliforms, following standard procedures.

The National System of Information of Hydric Resources (SNIRH) criteria to classify superficial water courses according to quality features for multiple applications (https://snirh.apambiente.pt) were used for the categorization of each physicochemical and microbiological parameter into three levels of water quality: bad, fair, and good (Table S1A). The overall quality of the sampling site was determined according to the worst category attributed to at least one parameter. Water samples from each of the fifteen sites were filtrated in triplicate through 0.45 μm pore membranes (Pall Life Sciences) and placed on mFC agar supplemented with 4 μg/mL of cefotaxime (Sigma-Aldrich, St. Louis, MI, USA). To determine the proportion of cefotaxime-resistant bacteria, mFC agar without antibiotics was used. Plates were incubated at 37 °C and counts were performed after 24 h. Individual cefotaxime-resistant colonies were purified and stored in 20% glycerol at −80 °C. 

### 2.3. Phylogenetic Affiliation

Individual colonies were used for amplification of a 16S rRNA gene fragment by PCR followed by sequence analysis. For that, a bacterial cell suspension of each isolate was prepared with 20 μL of dH_2_O. PCR amplification was carried out in a final volume of 25 μL containing 1 μL of cell suspension, 16.25 μL dH_2_O, 6.25 μL NZYTaq 2× Green Master Mix (2.5 mm MgCl_2_; 200 μm dNTPs; 1.25 U DNA polymerase) (NZYTech, Lisboa, Portugal), and 0.75 μL of each primer from a stock solution with a concentration of 10 μm. Conditions and primers (27F and 1492R) were used as described previously and detailed in Table S2. Positive and negative controls were included. The resulting amplicons were purified and Sanger sequenced at GATC, Konstanz, Germany. Nucleotide sequences were used for similarity searches against the GenBank database (https://www.ncbi.nlm.nih.gov/nucleotide/) using the BLASTn software (https://blast.ncbi.nlm.nih.gov/Blast.cgi). *Shigella* and *Escherichia* isolates were discriminated by culture in xylose lysine deoxycholate agar (XLD).

### 2.4. bla_CTX-M_ Screening, Genetic Environment, and Molecular Typing

All purified isolates were screened by PCR for the presence of the *bla*_CTX-M_ gene, using conditions and primers according to previous studies (Table S2). BOX-PCR and ERIC-PCR were used to assess clonality among all isolates carrying a *bla*_CTX-M_ gene, with primers and conditions as presented in Table S2. The final volumes for the reactions were 25 μL, which included 1 μL of cell suspension, 16.25 μL dH2O, 6.25 μL NZYTaq 2× Green Master Mix (NZYTech, Lisbon, Portugal), and 2 μL of primer for BOX-PCR (from a solution at 10 μm) or 1 μL of each primer for ERIC-PCR (from a solution at 50 μm). Banding profiles were analysed with GelCompar II (Applied Maths, Saint-Martens-Latem, Belgium). 

To determine the genetic context of the *bla*_CTX-M_ gene, PCR was used with primers specific for sequences known to be associated with the *bla*_CTX-M_ gene, namely IS*Ecp1* and IS*26* for the upstream region of the gene, and orf477 and IS*903* for the downstream region (Table S2), as previously described [40]. Positive controls were also used for every reaction. PCR products were then purified and sent for sequencing, and then assembled using BioEdit. Nucleotide and/or deduced amino acids sequences obtained were analysed against CARD (https://card.mcmaster.ca/analyze/blast), Genbank (https://blast.ncbi.nlm.nih.gov/Blast.cgi), and ISfinder (https://isfinder.biotoul.fr/blast.php) databases.

### 2.5. Antibiotic Susceptibility Testing of bla_CTX-M_ Positives

Susceptibility to antibiotics was tested by the disc diffusion method on Mueller–Hinton agar according to the procedure established by the European Committee on Antimicrobial Susceptibility Testing (EUCAST v11.0; https://eucast.org/). All *bla*_CTX-M_ positive isolates were tested against 16 antibiotics from 6 classes: beta-lactams (amoxicillin (AML, 10 μg), amoxicillin/clavulanic acid (AMC, 30 μg), piperacillin (PRL, 30 μg), piperacillin/tazobactam (TZP, 36 μg), ticarcillin (TIC, 75 μg), ticarcillin/clavulanic acid (TIM, 85 μg), cefotaxime (CTX, 5 μg), cefepime (FEP, 30 μg), ceftazidime (CAZ, 10 μg), aztreonam (ATM, 30 μg), and imipenem (IPM, 10 μg)), aminoglycosides (gentamicin (CN, 10 μg)), quinolones (ciprofloxacin (CIP, 5 μg)), tetracyclines (tetracycline (TE, 30 μg)), phenicols (chloramphenicol (C, 30 μg)), and the combination sulfamethoxazole/trimethoprim (SXT, 25 μg). After incubation at 37 °C for 18 h, isolates were classified as susceptible, resistant, or intermediate resistant. The quality control strain used was *E. coli* ATCC 25922.

### 2.6. Conjugation Assays

Conjugation assays were performed with the selected strains representing each *bla*_CTX-M_ gene variant identified. Mating tests were performed with the broth culture conjugation method using the rifampicin-resistant *E. coli* CV601 as the recipient strain [41]. Briefly, donors and recipient strains were mixed at a ratio of 1:1 in broth culture and grown for 24 h at 37 °C without agitation. Transconjugants were selected on plate count agar (PCA) containing cefotaxime (4 μg/mL) and rifampicin (100 μg/mL) and confirmed by BOX-PCR and by the presence of the *bla*_CTX-M_ gene. Susceptibility to antibiotics of the recipient strain and transconjugants was tested by the disc diffusion method on Mueller–Hinton agar, as described above.

## 3. Results

### 3.1. Water Quality Analysis and Cefotaxime-Resistant Bacteria Incidence

Water samples from all sites were classified as water with fair or poor quality, on both years (Figure 1, with P1 in 2018 representing the only sampling site with water samples classified with fair quality. In fact, the values registered for dissolved oxygen in all samples (except P1 in 2018) resulted in their classification as with poor water quality. Other parameters revealed the bad quality status of some sites such as phosphorus and nitrates, which were categorized as poor in nine and four sites, respectively, in at least one campaign (Figure 1). In some locations, the load of total and/or fecal coliforms increased in 2019, thus changing the categorization of these parameters from fair to bad (e.g., P7) or even from good to bad (e.g., P5). The highest number of poorly rated parameters was obtained at the P7 site in 2019 (six parameters).

**Figure 2:**
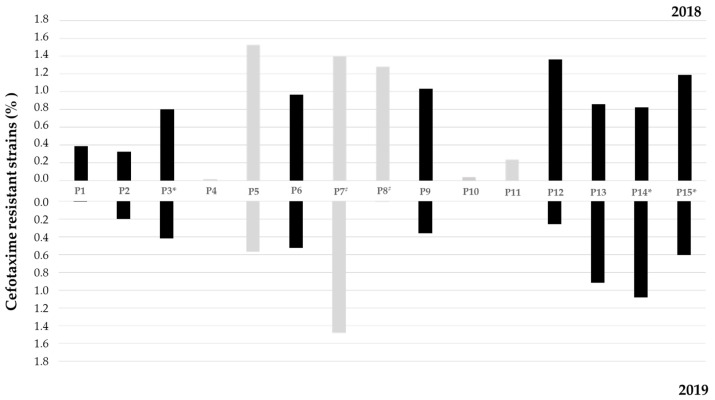
Proportion of cefotaxime-resistant bacteria determined in all sampling sites located in the Lis River from spring (P1) to mouth (P15) (P1–P3, P6, P9, P12–P15; black) and three affluents (P4/P5, P7/P8, and P10/P11; light grey); Sites located downstream WWTP and in the vicinities of pig farms are indicated by * and #, respectively.

The prevalence of cefotaxime-resistant bacteria (CTX^R^) varied from 0.02 to 1.53% in 2018 and from 0 to 1.48% in 2019 (Figure 2), with the highest values observed in sites located in Lis River affluents (P5 and P7) in 2018. In 2018, the proportion of CTX^R^ was higher than in 2019 in almost all sites and the occurrence of CTX^R^ bacteria was registered in all sites. While in 2018, site P5 presented the highest proportion of CTX^R^, in 2019, it was observed in site P7. In 2019, no CTX^R^ were detected in sites P4, P10, and P11, which also showed the lowest prevalence in 2018. 

### 3.2. Phylogenetic Affiliation of Cefotaxime-Resistant Isolates

One hundred and forty-seven CTX^R^ isolates were selected for further analysis, 91 isolated in 2018 and 56 in 2019. After 16S rRNA gene-based identification, a total of eight different genera were identified (Figure S1), five of which belonged to the *Enterobacteriaceae* family: *Escherichia*/*Shigella*, *Klebsiella*, *Citrobacter*, *Enterobacter*, and *Pantoea*. *Shigella*/*Escherichia* growth on xylose lysine deoxycholate agar (XLD) revealed that all isolates originated yellow colonies, confirming their affiliation to *Escherichia*. Non-*Enterobacteriaceae* genera were *Acinetobacter*, *Aeromonas*, and *Pseudomonas* (Figure S1). In 2018, all eight genera were found, whereas in the 2019 campaign, *Pantoea* isolates were not detected. Predominant genera varied between years, as shown in Figure S1. In 2018, *Acinetobacter* dominated, representing 35% of the total collection (*n* = 91), followed by *Escherichia* (32%), *Klebsiella* (12%), and *Pseudomonas* (10%). In 2019 (*n* = 56), *Acinetobacter* was represented by only one isolate, while *Klebsiella* and *Escherichia* were predominant (30% each). When comparing sampling sites (Figure S1), *Escherichia* was detected in 11 sites in 2018 and in 7 sites in 2019, and *Klebsiella* was detected in 5 sites in 2018 and 8 sites in 2019. Site P5 showed the highest diversity in 2018, with five genera isolated from this location, while in 2019, five different genera were retrieved from site P12 and four were retrieved from site P5.

### 3.3. Prevalence and Diversity of the bla_CTX-M_ Gene among the Cefotaxime-Resistant Isolates

**Figure 3:**
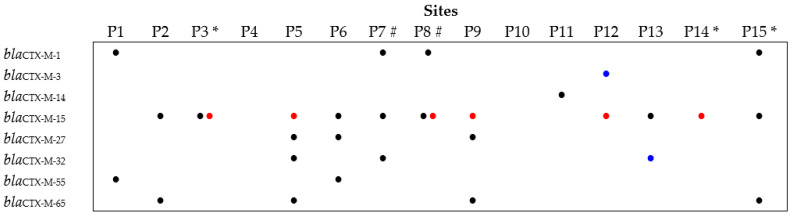
CTX-M encoding genes detected in the Lis riverine system. Black dots for *Escherichia* isolates, red for *Klebsiella*, and blue for *Citrobacter* or *Enterobacter*. Sites located downstream WWTP and in the vicinities of pig farms are indicated by * and #, respectively.

In 68 out of 147 isolates (46.3%), the presence of the *bla*_CTX-M_ gene was detected, distributed in four different *Enterobacteriaceae* genera, namely, *Escherichia* (*n* = 39), *Klebsiella* (*n* = 24), *Enterobacter* (*n* = 3), and *Citrobacter* (*n* = 2). These isolates were obtained from almost all sampled sites along the river and affluents (excluding sites P4 and P10) and in both years, as shown in Figure 3 and Table 1. Based on BOX and ERIC PCR-based analysis, one representative of each typing profile was selected from each site and year, resulting in a collection of 54 isolates. The analysis of the deduced amino acid sequences revealed that *bla*_CTX-M_ genes encoded seven different CTX-M variants, as shown in Table 1 and Figure 3.

Additionally, for 14 isolates (Table 1), it was not possible to determine the *bla*_CTX-M_ encoding variant. The CTX-M-15 encoding gene was the most prevalent, being present in 21 of all *bla*_CTX-M_ carriers (38.8%) (Table 1). The *bla*_CTX-M-1_, *bla*_CTX-M-32_, and *bla*_CTX-M-65_ genes were each present in 7.4% of the isolates. The *bla*_CTX-M-3 and_ *bla*_CTX-M-14_ genes showed the lowest prevalence, each being present in only one isolate. All variants were found in *Escherichia*, except *bla*_CTX-M-1_, with *bla*_CTX-M-15_ prevailing with 10 out of 32 isolates (31%), and the less frequent being *bla*_CTX-M-14_ in only one isolate. In *Klebsiella* isolates, only *bla*_CTX-M-15_ was detected. *bla*_CTX-M-3_ was present in only one *Enterobacter* isolate. The single *Citrobacter* isolate carried a *bla*_CTX-M-32_ gene.

**Figure 4:**
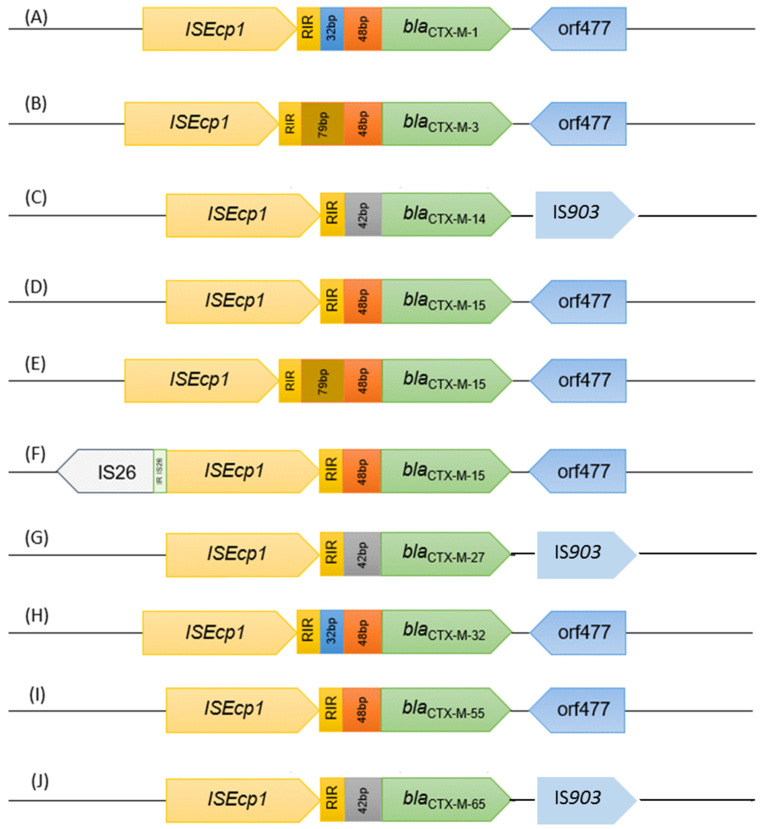
Genomic environments for the variants of the *bla*_CTX-M_ gene found in the isolates. Ten different environments were observed, and they are represented from (**A**–**J**).

The analysis of the regions flanking the different *bla*_CTX-M_ genes revealed the presence of 10 different genomic environments, as represented in Figure 4A–J. All shared the presence of the insertion sequence IS*Ecp1* in the upstream region of the gene, with variable distances to the gene (32 bp to 127 bp), mainly owing to the presence of different conserved regions previously reported [40,42,43] (Figure 4; Table 1). In the downstream region, orf477 and insertion sequence IS*903* were identified, as previously reported [40,42,43].

In regard to the diversity of CTX-M encoding genes detected along the Lis River (Figure 3), *bla*_CTX-M-15_ is well distributed along the river, found in 11 of the 15 sites sampled. Site 5 showed the highest diversity, with four different variants detected, followed by sites 6, 7, 9, and 15, with three different CTX-M encoding genes detected. *Klebsiella* isolates carrying *bla*_CTX-M-15_ genes were detected only in two sites: 3 (urban area, downstream a WWTP) and 8 (in the vicinity of swine farms).

### 3.4. Antibiotic Susceptibility Testing of bla_CTX-M_ Gene Carriers

Antibiotic susceptibility phenotypes were evaluated for all 54 isolates (Table 1). For analysis purposes, susceptibility profiles classified as intermediate were addressed as resistant. The overall results showed that isolates presented resistance levels below 60% to only four out of sixteen antibiotics, namely, piperacillin/tazobactam (38.8%), imipenem (3.7%), gentamicin (31.4%), and chloramphenicol (25.9%). Besides resistance to cefotaxime, the highest resistance rates were observed towards other beta-lactams, namely to cefepime, piperacillin, aztreonam, and ticarcillin, with 98% each, and 100% of the isolates were resistant to amoxicillin. Additionally, high resistance levels were observed for ciprofloxacin (79.6%), trimethoprim/sulfamethoxazole (72.2%), and tetracycline (62.9%).

Among *Escherichia* isolates, the lowest rates of resistance were observed towards imipenem (only 1 resistant), gentamicin (5 resistant isolates), and chloramphenicol (10 resistant isolates). *Klebsiella* isolates were highly susceptible to imipenem (1 resistant isolate), gentamicin (12 resistant isolates), tetracycline (9 resistant isolates), and chloramphenicol (3 resistant isolates). 

Bacteria are considered multi-resistant when they are non-susceptible to antibiotics included in three or more classes [44]. Hence, a total of 79.6% (corresponding to 43 isolates) showed multi-resistant phenotypes. All *Klebsiella* isolates were multi-resistant, as well as the single *Citrobacter* isolate, while among the *Escherichia* isolates, 75% showed a multi-resistant phenotype (*n* = 24). On the other hand, none of the *Enterobacter* isolates showed multi-resistance. Three out of the 54 isolates showed resistance to all tested antibiotic classes.

### 3.5. Conjugation Assays

Conjugation assays were performed for 18 isolates, as indicated in Table 1, representing the different variants identified and in the different genera. Under the conditions tested, we obtained transconjugants resistant to cefotaxime and rifampicin from 9 out of the 17 donor strains (Table 2). The antibiotic susceptibility tests revealed that all transconjugants were resistant to amoxicillin; ticarcillin; piperacillin; aztreonam; ceftazidime; and, as expected, cefotaxime. None showed resistance to imipenem, gentamicin, or chloramphenicol. Two presented a multi-resistance phenotype (E23T carrying a *bla*_CTX-M-27_ and K1T carrying a *bla*_CTX-M-15_).

## 4. Discussion

Rivers can act as reservoirs of multi-resistant bacteria, as they receive contaminants from all sources like WWTPs, industrial effluents, agricultural activities, hospital sewage, or animal production effluents [10,11,12,16]. All of these examples of contamination have been reported in the Lis River [34,35,36,37,38,45], rendering it of particular interest to the spread and evolution of antibiotic resistance in these ecosystems. 

When analyzing the water microbiological, physical, and chemical data, it was clear that the Lis River presents very poor water quality from the spring to the mouth, and in its affluents, as has been reported in previous studies [34,35,45]. For instance, sampling site P7, located in one of the Lis River affluents, globally presented the worst values observed in both years. The fact that this site is near several pig farms suggests a significant contribution from these sources of contamination. Swine slurry is a mixture of pig feces and urine with wastewater, and sometimes precipitation, which contains mainly suspended solids, nitrogen, phosphorous, and potassium [46]. Phosphorous levels were high across the river, but site P7 presented the highest values on both years. Furthermore, phosphorus levels in sites P3, P14, and P15 showed some concerning values in both years, which can relate to the location of these sampling sites downstream WWTPs.

In terms of abundance of *Enterobacteriaceae* resistant to cefotaxime along the river, site P7 also had the highest value among all sites. Besides the pollutants mentioned above, animal-production-derived effluents may contain different metals, antibiotics, antibiotic-resistant bacteria, and ARGs [47,48]. Moreover, it has been shown that ARGs from piggeries effluents can travel far by entering river courses, thus adding an additional public health risk [48]. In contrast, site P1 represents the spring of the Lis River, showing a small number of isolates resistant to cefotaxime, consistent with lower levels of pollution at this site. Focusing on the sites located in the river’s main course (P1, P2, P3, P6, P9, P12, P13, P14, and P15), it is noticeable that the percentage of cefotaxime-resistant *Enterobacteriaceae* displayed a tendency to increase along the river course, probably owing to an additive effect of different sources of contamination.

In 2019, all isolates retrieved from sites P7 and P8 carried the *bla*_CTX-M_ gene. Both sites are set at the Ribeira dos Milagres affluent, which is the part of the Lis River most affected by pollution [34]. In fact, the presence of the *bla*_CTX-M_ gene has been associated with high levels of anthropogenic influence [40].

*Escherichia* and *Klebsiella* together represented 93% of *bla*_CTX-M_ carriers. These two genera include clinically relevant pathogens, and in this case, are particularly critical owing to their ESBL-producing and multi-resistance traits. Therefore, their frequent occurrence in the Lis River is alarming, as these waters can easily reach the population. Risks to human health arise from the fact that the water from the Lis River is frequently used for irrigation, fishing, and leisure activities, facilitating the contact of humans with resistant bacteria [49]. Previous studies confirmed the transfer of antibiotic-resistant bacteria from irrigation water to vegetables that are consumed raw [41]. Moreover, the transfer of cefotaxime-resistant bacteria from water to humans during leisure activities has been confirmed [50]. Infections caused by resistant strains from these groups of bacteria are growing every year across all continents [17,25]. They are often found in clinical environments (i.e., hospitals) and it is becoming harder to respond to these infections, thus there is an urgent need for new antibiotics for dealing with infections caused by members of these groups, according to the World Health Organization [51].

When analyzing clonality among our collection, we noticed that, even though most of the isolates had unique profiles, a few isolates collected in different sites presented identical profiles. This may indicate that these strains can survive in this environment for some period, being transported along the river. If their genetic determinants of resistance are located in mobile elements, their persistence may promote gene transfer to other bacteria. Furthermore, clonal isolates were also collected in different years, suggesting that they may derive from a persistent contamination source. However, further studies are needed to confirm this hypothesis.

The *bla*_CTX-M_ genes are well known for being associated with other antibiotic resistance genes. This derives from the fact that *bla*_CTX-M_ genes are often located in conjugative plasmids that harbor resistance genes to fluoroquinolones, aminoglycosides, and sulfonamides [28]. Keeping this in mind, it is not surprising that a total of 79.6% of the isolates were classified as multi-resistant. Despite the low rate of resistance to imipenem (4%), it is still very concerning. Carbapenems are used as last resource antibiotics to treat *Enterobacteriaceae* infections [20], and their increased prescription is promoted by the growing prevalence of ESBL producers. Ultimately, this may result in higher numbers of carbapenemase-producing *Enterobacteriaceae* infections. Worryingly, a recent study showed that the Lis River is in fact contaminated with carbapenem-resistant *Enterobacteriaceae* [38,39], carrying plasmids that harbour different genetic determinants of resistance, identical to those already described in clinical settings, namely, *bla*_NDM_, *bla*_KPC_, and *bla*_GES_. Furthermore, through qPCR analysis, *bla*_CTX-M_ was detected in all sampling sites and showed a significant increase in the overall gene abundance over one year [39].

In contrast, high resistance rates to tetracycline and trimethoprim/sulfamethoxazole (63% and 72%, respectively) are not surprising as these are antibiotics commonly used in animal production, a common practice in this region. Furthermore, sulfamethoxazole was one of the main antibiotics detected in the Lis River water by Paiga and co-authors [37].

Though a strategy based on culture-dependent methods can be seen as a limitation, to characterize in detail these resistance mechanisms, this approach is fundamental and quite relevant in epidemiological terms. Higher *bla*_CTX-M_ gene diversity was observed in sites located in urban areas (e.g., P5 and P6) or in the proximity of pig farms (e.g., P7 and P9). Interestingly, the majority of *Klebsiella* isolates carrying *bla*_CTX-M_ genes were detected in sites located in urban areas (P3, P5, and P6), next to WWTPs (P3 and P14), or in the proximity of pig farms (P8 and P9). All CTX-M variants identified in this study have been previously identified in Portugal in ESBL producers isolated not only in clinical, but also in environmental samples, such as, for example, from companion [52,53] and wild animals [54], retail meat [55], and wastewater-treated effluents or rivers [14,15,40,56,57]. Most variants were also previously found in rivers in Portugal [40], although, to the best of our knowledge, this is the first report showing the presence of *bla*_CTX-M-65_ in a Portuguese river. This variant has been identified worldwide in various sources such as food-related products, wild and companion animals, or aquatic systems [58,59,60], but less commonly in European countries. A recent study in Portugal reported the presence of *E. coli* isolates retrieved from retail meat carrying *bla*_CTX-M-65_ [55]. In fact, the genomic context was identical to that characterized in CTX-M-65-producing *E. coli* isolates obtained in this study; that is, with *bla*_CTX-M-65_ flanked by IS*Ecp1* (upstream) and IS*903* (downstream) [55].

In this study, *bla*_CTX-M-15_ stood out as the most prevalent gene variant, detected in 52.5% of isolates. The *bla*_CTX-M-15_ was identified for the first time in 1980s and, since then, has become the most prevalent *bla*_CTX-M_ gene variant worldwide, in both hospitals and the environment [40,61,62,63,64]. It was also the main variant found in Portuguese aquatic systems [25,39] and WWTPs, including in treated effluents [14,15,57].

The results obtained here can be interpreted as a global alert for aquatic systems of similar characteristics. The Lis hydrographic basin gathers several features relevant for the study of antibiotic resistance dissemination in aquatic systems. On one hand, surface water use is quite relevant in this region, with agriculture being a key economic activity. On the other hand, this hydrographic basin encompasses an area of both urban and rural characteristics. Several anthropogenic impacts are identified along the river’s main course and its tributaries, with wastewater treatment plants and pig farms potentially constituting major contributors to the global low water quality that has been reported here in the last decades [34,35,36,37,38,39]. Thus, information is needed to determine the main sources of contamination and thus implement adequate management plans. For instance, the data provided here can give an indication of the efficiency of wastewater treatment, thus helping to support necessary changes. Informed measures are generally more economically sustainable because they are more effective.

The overall results highlight the relevance of similar studies to the global knowledge on the prevalence and dispersion of antibiotic resistance in correlation with water pollution and anthropogenic impacts. Water scarcity and growing worldwide population has brought attention to the need to provide more water through alternative approaches such as, for example, desalination and water reuse, as well as for water quality improvement in global water resources [2]. Water resource management plans and policy decisions usually envision water quality evaluation based on a few parameters, but do not consider antibiotic-resistant bacteria and antibiotic resistance determinants, despite their public health relevance. Hence, our work also brings attention to the need to establish prompt municipal warning systems that include monitoring of water pollutants (including parameters related to antibiotic resistance), identifying the main contributors to decreases in water quality, and following water management plans.

## 5. Conclusions

As expected, the data from this work expose the high levels of pollution in the Lis River, and suggest that pig farms represent a major source of pollution in this region. However, agricultural run-offs, wastewater from hospitals, and WWTPs should also be considered. Water samples from highly polluted sampling sites also demonstrated a higher prevalence of CTX-M producers of high clinical relevance and that exhibit multi-resistance phenotypes. Altogether, these findings constitute concerning factors to natural environments as well as public health. Further research is necessary to evaluate the actual size of the risk to public health posed by the different uses that are given to this water system. For instance, agricultural products irrigated with this water or aquatic animals used for human consumption should be evaluated, as well as recreational areas, by inspecting not only water, but also sediments and beach sand. Additionally, these studies could be complemented with data obtained through culture-independent strategies. 

## Figures and Tables

**Table 1:** Description of *bla*_CTX-M_ carriers isolated in the Lis River concerning isolation year and location, identification, antibiotic susceptibility profiles (black—resistant, grey—intermediate resistance, white—susceptible), and *bla*_CTX-M_ variant detected and genomic context.

Affiliation	Campaign Year	Site	Strain ^a^	Antibiotic Susceptibility ^b^																*bla*_CTX-M variant_^c^	Genetic Environment of *bla*_CTX-M_				
				AML	AMC	PRL	TZP	TIC	TIM	ATM	CAZ	CTX	FEP	IPM	CN	CIP	TE	C	SXT		IS*Ecp1*	IS*26*	orf477	IS*903*	In Figure 3
***Escherichia***	2018	1	E1																	nd	+			+	nd
		1	E2 *																	−1	+		+		A
		5	E3 *																	−32	+		+		H
		5	E4																	−65	+			+	J
		7	E5 *																	−1	+		+		A
		8	E6																	−1	+		+		A
		8	E7 *																	−15	+		+		D
		9	E8 *																	−65	+			+	J
		9	E9																	nd	+		+		nd
		11	E10 *																	−14	+			+	C
		13	E11																	nd	+		+		nd
		13	E12																	−15	+		+		E
		15	E13 *																	−15	+		+		D
		1	E14																	nd	+				nd
		1	E15																	−55	+		+		I
		2	E16																	−65	+			+	J
		3	E17																	−15	+		+		D
		3	E18																	−15	+		+		D
		5	E19 *																	−32	+		+		H
		6	E20																	−15	+	+	+		F
	2019	2	E21																	−15	+		+		D
		5	E22 *																	−27	+			+	G
		6	E23 *																	−27	+			+	G
		6	E24 *																	−55	+		+		I
		7	E25																	nd	+				nd
		7	E26																	−32	+		+		H
		7	E27																	−15	+		+		D
		8	E28																	−15	+		+		D
		9	E29																	−27	+			+	G
		15	E30																	−15	+		+		D
		15	E31 *																	−65	+			+	J
		15	E32																	−1	+		+		A
***Klebsiella***	2018	5	K1 *																	−15	+		+		D
		8	K2																	nd	+		+		nd
		8	K3																	−15	+		+		D
		9	K4																	−15	+		+		D
		14	K5																	−15	+		+		D
		14	K6 *																	−15	+		+		D
	2019	3	K7																	−15	+		+		D
		3	K8 *																	−15	+		+		D
		3	K9																	nd	+		+		nd
		3	K10																	−15	+		+		D
		5	K11																	−15	+		+		D
		6	K12																	nd	+		+		nd
		9	K13																	nd	+		+		nd
		9	K14																	nd	+		+		nd
		12	K15																	−15	+	+	+		F
		13	K16																	nd	+		+		nd
		14	K17																	nd	+		+		nd
		14	K18																	−15	+		+		D
***Citrobacter***	2018	12	C1																	nd		+			nd
	2019	12	C2 *																	−3	+		+		B
		12	C3 *																	nd	+		+		nd
***Enterobacter***		13	C4 *																	−32	+		+		H

^a^ AML—amoxicillin, AMC—amoxicillin/clavulanic acid, PRL—piperacillin, TZP—piperacillin/tazobactam, TIC—ticarcillin, TIM—ticarcillin/clavulanic acid, ATM—aztreonam, CAZ—ceftazidime, CTX—cefotaxime, FEP—cefepime, IPM—imipenem, CN—gentamicin, CIP—ciprofloxacin, TE—tetracycline, C—chloramphenicol, SXT—sulfamethoxazole/trimethoprim; ^b^ strains used in conjugation assays are marked with *; ^c^ nd: not determined.

**Table 2:** Antibiotic susceptibility profiles of donor and transconjugant strains carrying *bla_CTX-M_* genes and recipient strain *E. coli* CV601 (black—resistant, grey—intermediate resistance, white—susceptible).

		Antibiotic Susceptibility															
Donor/Transconjugant Strains and *bla*_CTX-M_ Gene		AML	AMC	PRL	TZP	TIC	TIM	ATM	CAZ	CTX	FEP	IPM	CN	CIP	TE	C	SXT
*Escherichia* E3:: *bla*_CTX-M-32_	E3D																
	E3T																
*Escherichia* E5:: *bla*_CTX-M-1_	E5																
	E5T																
*Escherichia* E19:: *bla*_CTX-M-32_	E19																
	E19T																
*Escherichia* E23:: *bla*_CTX-M-27_	E23																
	E23T																
*Escherichia* E24:: *bla*_CTX-M-55_	E24																
	E24T																
*Klebsiella* K1:: *bla*_CTX-M-15_	K1																
	K1T																
*Klebsiella* K6:: *bla*_CTX-M-15_	K6																
	K6T																
*Citrobacter* C3:: *bla*_CTX-M-nd_	C3																
	C3T																
*Citrobacter* C4:: *bla*_CTX-M-32_	C4																
	C4T																
Recipient strain *E. coli* CV601	-																

AML—amoxicillin, AMC—amoxicillin/clavulanic acid, PRL—piperacillin, TZP—piperacillin/tazobactam, TIC—ticarcillin, TIM—ticarcillin/clavulanic acid, ATM—aztreonam, CAZ—ceftazidime, CTX—cefotaxime, FEP—cefepime, IPM—imipenem, CN—gentamicin, CIP—ciprofloxacin, TE—tetracycline, C—chloramphenicol, SXT—sulfamethoxazole/trimethoprim.

## Data Availability

Not applicable.

## Supplementary Materials

The following supporting information can be downloaded at: https://www.mdpi.com/article/10.3390/ijerph191911858/s1, Table S1: Physical, chemical, and microbiological parameters measured to classify water quality status as poor, fair, or good (A), determined in 2018 and 2019 sampling campaigns (B) and overall classification. The classification considers parameters and maximum recommended values established by the Portuguese government (adapted from https://snirh.apambiente.pt/snirh/_dadossintese/qualidadeanuario/boletim/tabela_classes.php); Table S2. Primers used in this study; Figure S1. Genus-level affiliation of isolates retrieved from each site (1 to 15) in 2018 (A) and 2019 (B), with the number of isolates for each genus represented in bars. References [39,42,65,66,67,68] are cited in the supplementary materials.
