# Supplementary material for: CTX-M-Producing Bacteria Isolated from a Highly Polluted River System in Portugal
Source: Int J Environ Res Public Health. 2022 Sep 20;19(19):11858. doi: 10.3390/ijerph191911858 (PMC9565674; doi:10.3390/ijerph191911858)
Supplement: Supplementary file 1 [file ijerph-19-11858-s001.zip › ijerph-1846740-supplementary.pdf]

Supplemental material

CTX-M-producing bacteria isolated from a highly polluted river system in Portugal

Marta Tacão [1#], José Laço [2#], Pedro Teixeira [3] and Isabel Henriques [4*]

[1] CESAM and Biology Department, University of Aveiro, Aveiro, Portugal
[2] University of Coimbra, Department of Life Sciences, Coimbra, Portugal
[3] CESAM and Biology Department, University of Aveiro, Aveiro, Portugal
[4] University of Coimbra, Department of Life Sciences and CFE, Coimbra, Portugal
* Correspondence: isabel.henriques@uc.pt;
# These authors contributed equally to this work

Contents:

Table S1

Table S2

Figure S1

Table S1. Physical, chemical and microbiological parameters measured to classify water quality status as poor, fair or good (A), determined in 2018 and 2019 sampling campaigns (B) and overall classification. The classification considers parameters and maximum recommended values established by the Portuguese government (adapted from https://snirh.apambiente.pt/snirh/_dadossintese/qualidadeanuario/boletim/tabela_classes.php).

(A)

	**Poor**	**Fair**	**Good**
**pH**	< 6 or > 9	-	6 - 9
**Conductivity (μS/cm)**	> 1500	1000-1500	≤ 1000
**Dissolved Oxygen (%)**	≤ 30	30-70	≥ 70
**Biochemical Oxygen Demand (mg/l)**	> 8	5-8	≤ 5
**Nitrates (mg/l)**	>50	25-50	≤ 25
**Phosphorus (mg/l)**	> 0.4	0.25-0.4	≤ 0.25
**Faecal Coliforms CFU/100ml**	> 20000	2000-20000	≤ 2000
**Total Coliforms CFU/100ml**	> 50000	5000-50000	≤ 5000
***Enterococcus* CFU/100ml**	> 20000	2000-20000	≤ 2000

(B)

		Temperature ºC	pH	Conductivity µS/cm	Dissolved Oxygen (%)	Biochemical Oxygen Demand mg/l	Nitrates mg/l	Phosphorus mg/l	Faecal Coliforms CFU/100ml	Total Coliforms CFU/100ml	*Enterococcus* CFU/100ml	Classification
**P1**	**2018**	15.9	7.4	574	42.3	< 3	6.8	< 0.05	190	3900	210	
	**2019**	18.7	7.6	626	23.5	< 3	12.0	< 0.05	43000	50000	53000	
**P2**	**2018**	14.2	8.1	524	19.5	< 3	< 1.0	< 0.05	380	5000	7100	
	**2019**	18.1	7.8	587	15.7	< 3	9.0	< 0.05	13000	35000	2300	
**P3**	**2018**	14.7	7.9	416	19.6	6.0	15.0	0.60	7700	8700	7100	
	**2019**	17.4	7.7	549	13.5	< 3	19.0	0.52	9800	43000	3500	
**P4**	**2018**	13.3	8.1	654	23.1	3.0	12.0	0.24	4000	7100	5900	
	**2019**	17.2	7.4	1000	10.8	< 3	7.0	0.17	290	6200	400	
**P5**	**2018**	13.8	8.0	654	23.2	< 3	16.0	0.35	2000	4900	7100	
	**2019**	18.1	7.6	934	13.4	< 3	17.0	0.27	60000	99000	5300	
**P6**	**2018**	14.7	7.9	614	17.5	4.0	17.0	0.52	6900	9100	9100	
	**2019**	18.6	7.6	1420	11.2	<3	23.0	0.34	25000	40000	1900	
**P7**	**2018**	10.8	7.8	502	21.1	7.0	68.0	1.93	4000	5500	900	
	**2019**	19.0	7.3	555	11.5	17.0	86.0	3.46	34000	100000	13000	
**P8**	**2018**	13.9	7.8	254	20.6	3.0	27.0	0.66	7900	9300	8100	
	**2019**	16.5	7.6	484	12.4	< 3	41.0	0.50	320	6600	250	
**P9**	**2018**	14.0	8.2	578	21.4	< 3	17.0	0.17	4200	8700	1500	
	**2019**	19.4	7.5	1030	15.0	< 3	68.0	0.48	8600	21000	600	
**P10**	**2018**	13.6	7.5	756	9.7	< 3	52.0	1.13	1900	2500	2000	
	**2019**	19.0	7.6	957	10.6	3.0	35.0	0.41	150	760	200	
**P11**	**2018**	14.3	7.6	544	13.3	5.0	38.0	0.52	6000	9300	3400	
	**2019**	19.8	7.6	980	8.7	6.0	11.0	0.32	120	36000	40	
**P12**	**2018**	14.4	8.0	554	19.3	< 3	23.0	0.34	3900	7500	2200	
	**2019**	19.3	7.5	770	13.2	< 3	29.0	0.35	1300	34000	310	
**P13**	**2018**	14.4	8.0	565	18.7	< 3	23.0	0.39	5900	7900	1800	
	**2019**	20.0	7.6	859	14.2	< 3	40.0	0.31	1700	23000	110	
**P14**	**2018**	15.9	7.8	592	22.3	< 3	23.0	0.60	6200	9500	1800	
	**2019**	20.3	7.2	1040	9.7	< 3	22.0	0.58	1100	23000	2300	
**P15**	**2018**	14.9	7.8	624	24.0	< 3	20.0	0.40	4200	8700	1700	
	**2019**	19.4	7.6	1370	8.8	< 3	99.0	0.56	11000	52000	210	

Table S2: Primers used in this study

Target	Amplicon size (bp)	Primers sequence (5′-3′)	Reference
**16S rRNA gene**	1467	27F: AGAGTTTGATCCTGGCTCAG	Lane, D. J. (1991)
		1492R: GGYTACCTTGTTAACGACTT	
***bla*$_{CTX-M}$**	538	CTX_F: GTGCAGTACCAGTAAAGTTATGG	Henriques et al.. (2006)
		CTX_R: CGCAATATCATTGGTGGTGCC	
**BOX element**	Variable	BOX A1R: CTACGGCAAGGCGACGCTGACG	Versalovic et al. (1991)
**ERIC element**	Variable	ERIC1: AAGTAAGTGACTGGGGTGAGC	
		ERIC2: ATGTAAGCTCCTGGGGATTCAC	
**IS*Ecp1***	Variable	TTCAAAAAGCATAATCAAAGCC	Eckert et al. (2006)
**IS26**		AGCGGTAAATCGTGGAGTGA	Saladin et al. (2002)
**Orf477**		GGTGGCATAATTTTTGAAGT	Eckert et al. (2006)
**IS*903***		CATCATCCAGCCAGAAAGTT	Eckert et al. (2006)

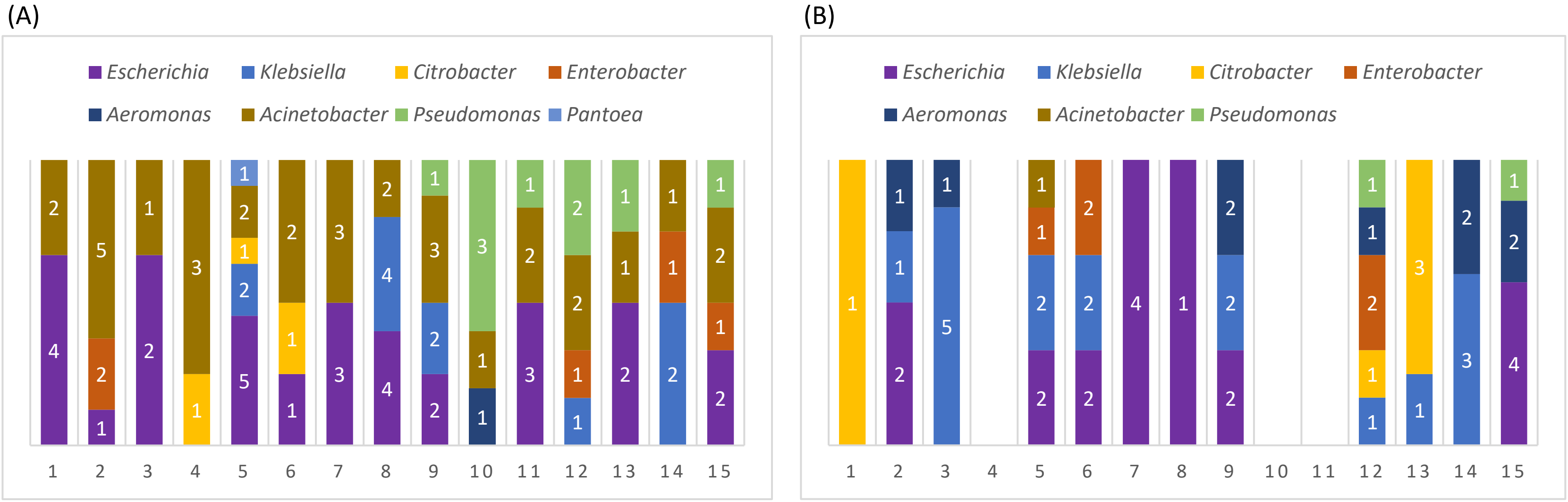

Figure S1. Genus-level affiliation of isolates retrieved from each site (1 to 15) in 2018 (A) and 2019 (B), with the number of isolates for each genus represented in bars.

REFERENCES

Teixeira, P.; Tacão, M.; Henriques, I. Occurrence and distribution of Carbapenem-resistant *Enterobacterales* and carbapenemase genes along a highly polluted hydrographic basin. *Environ Pollut* **2022**, 300, 118958

Lane, D.J. 16S/23S rRNA Sequencing. In: Stackebrandt, E. and Goodfellow, M., Eds., Nucleic Acid Techniques in Bacterial Systematic, John Wiley and Sons, New York **1991**, 115-175.

Henriques, I. S.; Fonseca, F.; Alves, A.; Saavedra, M. J.; Correia, A. Occurrence and diversity of integrons and β-lactamase genes among ampicillin-resistant isolates from estuarine waters. *Res Microbiol* **2006**, 157(10), 938–947.

Versalovic, J.; Koeuth, T.; Lupski, R. Distribution of repetitive DNA sequences in eubacteria and application to fingerprinting of bacterial genomes. *Nucleic Acids Res* **1991**, 19(24), 6823–6831

Eckert, C.; Gautier, V.; Arlet, G. DNA sequence analysis of the genetic environment of various blaCTX-M genes. *J Antimicrob Chemother* **2006**, 57(1), 14–23.

Saladin, M.; Cao, V.T.; Lambert, T.; Donay, J.L.; Herrmann, J.L.; Ould-Hocine, Z.; Verdet, C.; Delisle F.; Philippon A.; Arlet, G. Diversity of CTX-M beta-lactamases and their promoter regions from Enterobacteriaceae isolated in three Parisian hospitals. *FEMS Microbiol Lett* **2002**, 209, 161-168.
